# Supplementary material for: Tick-Borne Encephalitis Specific Lymphocyte Response after Allogeneic Hematopoietic Stem Cell Transplantation Predicts Humoral Immunity after Vaccination
Source: Vaccines (Basel). 2021 Aug 15;9(8):908. doi: 10.3390/vaccines9080908 (PMC8402406; doi:10.3390/vaccines9080908)

**Table S1.** Antibodies used for cytokine detection with Luminex system (Luminex 100SI, Biomedica, Vienna, Austria)

| Luminex Microspheres Bead region | Standard | | | Antibody specificity | Capture Antibody | | | Biotinylated Detection Antibody | | |
|---|---|---|---|---|---|---|---|---|---|---|
| | Standard entity | Catalog number | Company | | Clone | Catalog number | Company | Clone | Catalog number | Company |
| 006 | IL-2 | 200-02-10μg | Peprotech | IL-2 | MQ1-17H12 | 14-7029-85 | eBioscience | Polyclonal | 13-7028-85 | eBioscience |
| 021 | IL-10 | 200-10-2μg | Peprotech | IL-10 | JES3-9D7 | 14-7108-85 | eBioscience | JES3-12G8 | 13-7109-85 | eBioscience |
| 035 | IL-13 | 200-13-2μg | Peprotech | IL-13 | JES10-5A2 | 501902 | Biolegend | Poly5020 | 502001 | Biolegend |
| 048 | GM-CSF | 572902 | Biolegend | GM-CSF | BVD2-23B6 | 502202 | Biolegend | BVD2-21C11 | 502304 | Biolegend |
| 074 | IFNγ | 300-02-20μg | Peprotech | IFNγ | MD-1 | 507502 | Biolegend | 4S.B3 | 502504 | Biolegend |
| 041 | TNFα | 300-01A-10μg | Peprotech | TNFα | MAb1 | 502802 | Biolegend | MAb11 | 502904 | Biolegend |

**Table S2:** The following table shows immune reconstitution data from all patients at baseline (= before first vaccination). Lymphocyte subgroups were differentiated by FACS (fluorescence-activated cell scanning) from unstimulated cells.

| **Patient** | **Lymphocytes** | **T-lymphocytes** | **CD4+ cells** | **CD8+ cells** | **CD4+ memory** | **CD4+ naive** | **CD8+ memory** | **CD8+ naive** | **B-Lymphocytes** | **class switched** | **non-class switched** | **naive** | **immature** | **transitional** | **plasmablasts** |
|---|---|---|---|---|---|---|---|---|---|---|---|---|---|---|---|
| *Normal range* | *1000-4000* | *m 590-2000 f 770-2200* | *m 360-1350 f 310-1570* | *280-1000* | *35-89%* | *12-78%* | *8-29%* | *8-29%* | *110-530* | *7-39%* | *5-57%* | *NA* | *NA* | *NA* | *NA* |
| Unit | absolut/µl | absolut/µl | absolut/µl | absolut/µl | %CD4 | %CD4 | %CD8 | %CD8 | absolut/µl | %CD19 | %CD19 | %CD19 | %CD19 | %CD19 | %CD19 |
| **1** | 1568 | 1192 | 470 | 580 | 56.2 | 29.9 | 10.5 | 60.2 | 110 | 5.5 | 5.6 | 83.8 | 1.1 | 4.6 | 10.3 |
| **2** | 1376 | 716 | 358 | 330 | 21.8 | 42.9 | 2.9 | 82.0 | 592 | 0.7 | 4.3 | 92 | 0.3 | 7.2 | 38.2 |
| **3** | 1050 | 494 | 231 | 200 | 38.2 | 39.7 | 20.5 | 49.8 | 315 | 2.9 | 25.2 | 68.9 | 2.8 | 3.8 | 15.3 |
| **4** | 1026 | 523 | 257 | 267 | 69.3 | 18.1 | 54.3 | 22.0 | 380 | 1.1 | 4.5 | 79.7 | 1.5 | 2.2 | 28.7 |
| **5** | 2592 | 1555 | 337 | 1192 | 88.4 | 1.93 | 20.8 | 20.8 | 389 | 4.9 | 12 | 76.2 | 0.8 | 15.8 | 11.1 |
| **6** | 1840 | 1214 | 442 | 626 | 49.4 | 30.5 | 9.7 | 59.2 | 515 | 1 | 4.1 | 90.4 | 0.8 | 4.6 | 16.9 |
| **7** | 1430 | 1115 | 315 | 758 | 62.7 | 21.7 | 14.8 | 51.1 | 129 | 0.3 | 2.5 | 95.3 | 3.7 | 31.5 | 24.7 |
| **8** | 1204 | 999 | 518 | 482 | 40.5 | 23.0 | 22.5 | 30.8 | 157 | 3.9 | 16.6 | 70 | 1.6 | 14 | 7 |
| **9** | 1280 | 576 | 294 | 282 | 48.1 | 3.6 | 29.6 | 12.8 | 525 | 4.1 | 8 | 84.6 | 1.2 | 14 | 10.2 |
| **10** | 1700 | 867 | 527 | 340 | 67.4 | 12.9 | 21.1 | 41.9 | 442 | 0.4 | 2 | 94.3 | 3.6 | 38.9 | 1.3 |
| **11** | 1800 | 648 | 396 | 216 | - | - | 22.1 | 52.9 | 810 | 1.4 | 2.2 | 92.7 | 4.1 | 57.7 | 3.1 |
| **12** | 1178 | 766 | 306 | 424 | 66.3 | 20.2 | 31.9 | 30.9 | 224 | 5.6 | 6.3 | 76.7 | 1.3 | 72.7 | 4.4 |
| **13** | 1782 | 1568 | 321 | 1212 | 60.7 | 17.4 | 21 | 33.1 | 71 | 16.1 | 10.9 | 66.7 | 1.5 | 23.3 | 3.8 |
| **14** | 3224 | 1096 | 387 | 645 | 86.8 | 6.5 | 70.9 | 6.7 | 1709 | 4.9 | 1 | 86.4 | 0.9 | 65.9 | 17.1 |
| **15** | 2400 | 2232 | 72 | 2160 | 90.5 | 2.5 | 26.2 | 25.5 | 24 | 7.8 | 9.2 | 68 | 5.8 | 24.9 | 7.1 |
| **16** | 1634 | 1226 | 621 | 458 | 49.6 | 39.9 | 18.9 | 60.1 | 327 | 3.3 | 7.1 | 82 | 1.1 | 39.5 | 5.2 |
| **17** | 1972 | 1499 | 394 | 1124 | 65.2 | 15.2 | 4.4 | 62.6 | 434 | 1.7 | 3.7 | 90.1 | 1.9 | 61.8 | 10.1 |

**Table S3:** The following table shows immune reconstitution data from all patients at two different time points during the study period: 12 weeks after first vaccination and 9 to 11 months after baseline at the time of third vaccination. Lymphocyte subgroups were differentiated by FACS (fluorescence-activated cell scanning) from unstimulated cells.

| **Patient** | **Lymphocytes** | **Lymphocytes** | **T-lymphocytes** | **T-lymphocytes** | **CD4+ cells** | **CD4+ cells** | **CD8+ cells** | **CD8+ cells** | **B-lymphocytes** | **B-lymphocytes** |
|---|---|---|---|---|---|---|---|---|---|---|
| *Normal range* | *1000-4000* | *1000-4000* | *m 590-2000 f 770-2200* | *m 590-2000 f 770-2200* | *m 360-1350 f 310-1570* | *m 360-1350 f 310-1570* | *280-1000* | *280-1000* | *110-530* | *110-530* |
| Unit | absolut/µl | absolut/µl | absolut/µl | absolut/µl | absolut/µl | absolut/µl | absolut/µl | absolut/µl | absolut/µl | absolut/µl |
| Time point | 12 weeks post Baseline | 9 to 11 months post Baseline | 12 weeks post Baseline | 9 to 11 months post Baseline | 12 weeks post Baseline | 9 to 11 months post Baseline | 12 weeks post Baseline | 9 to 11 months post Baseline | 12 weeks post Baseline | 9 to 11 months post Baseline |
| **1** | 1431 | 1647 | 1116 | 1235 | 529 | 609 | 501 | 544 | 186 | 231 |
| **2** | 1273 | 1862 | 586 | 726 | 306 | 354 | 267 | 317 | 458 | 1005 |
| **3** | 1188 | 1560 | 594 | 827 | 273 | 359 | 249 | 374 | 261 | 390 |
| **4** | 1482 | 1539 | 904 | 877 | 415 | 431 | 489 | 431 | 430 | 477 |
| **5** | 2640 | 2109 | 1558 | 1202 | 449 | 380 | 1030 | 801 | 528 | 506 |
| **6** | 2184 | 2310 | 1310 | 1247 | 546 | 531 | 655 | 624 | 743 | 901 |
| **7** | 2550 | - | 1836 | - | 485 | - | 1275 | - | 459 | - |
| **8** | 1836 | 2021 | 1542 | 1556 | 771 | 869 | 771 | 687 | 239 | 404 |
| **9** | 1829 | 1464 | 750 | 498 | 311 | 264 | 439 | 220 | 695 | 703 |
| **10** | 1892 | - | 946 | - | 530 | - | 416 | - | 359 | - |
| **11** | 1798 | - | 575 | - | 342 | - | 216 | - | 971 | - |
| **12** | 1150 | - | 725 | - | 311 | - | 403 | - | 207 | - |
| **13** | 2163 | 2937 | 1925 | 2526 | 346 | 529 | 1514 | 1968 | 87 | 206 |
| **14** | 3509 | 3900 | 1263 | 1716 | 561 | 702 | 667 | 936 | 1790 | 1365 |
| **15** | 4230 | 2668 | 3807 | 1948 | 254 | 213 | 3596 | 1734 | 254 | 560 |
| **16** | 3569 | 2352 | 2356 | 1740 | 1356 | 917 | 785 | 612 | 1106 | 517 |
| **17** | 4410 | 2240 | 3396 | 1523 | 706 | 403 | 2690 | 1120 | 838 | 672 |

**Table S4.** The following table shows descriptive statistics (Mean, Standard Deviation, Median, 1st and 3rd Quantile, Minimum, Maximum and sample size N) for cellular data separately for patients and controls as well as overall. The p-values give the result for the Wilcoxon-Test comparing patients with controls. Lymphocyte proliferation has been standardized based on unstimulated control samples and is shown as stimulation index (SI).

Time: BL – Baseline; 2nd – after 2nd vaccination; 3rd – after 3rd vaccination. TBE: tick borne encephalitis antigen; CEFT: CMV, EBV, influenza virus and Clostridium tetani antigen mix; PMA/Iono: phorphole myristate acetate plus ionomycin.

| Variable | Time | Group | Mean | SD | Median | Q1 | Q3 | Min | Max | N | p-value |
|---|---|---|---|---|---|---|---|---|---|---|---|
| Age | BL | Control | 32 | 12.81 | 29.5 | 22.75 | 34.25 | 21 | 60 | 8 | 0.3808 |
| | | Patient | 38 | 14.34 | 31 | 26 | 53 | 22 | 61 | 17 | |
| | | overall | 36.08 | 13.9 | 31 | 26 | 47 | 21 | 61 | 25 | |
| BMI | BL | Control | 22.48 | 2.86 | 21.65 | 20.75 | 24.65 | 18.5 | 26.6 | 8 | 0.0359 |
| | | Patient | 27.48 | 6.44 | 26.9 | 23.1 | 29.9 | 19.1 | 43.1 | 17 | |
| | | overall | 25.88 | 5.98 | 24.2 | 22.2 | 28.4 | 18.5 | 43.1 | 25 | |
| TBE | BL | Control | 1.29 | 0.67 | 0.98 | 0.88 | 1.53 | 0.61 | 2.37 | 8 | 0.0019 |
| Proliferation | | Patient | 13.29 | 18.2 | 4.15 | 1.45 | 24.34 | 1.02 | 69.7 | 17 | |
| | | overall | 9.45 | 15.92 | 2.22 | 1.04 | 9.42 | 0.61 | 69.7 | 25 | |
| | 2nd | Control | 10.25 | 8.37 | 8.3 | 4.29 | 12.92 | 1.75 | 23.93 | 8 | 0.9773 |
| | | Patient | 18.33 | 22.08 | 8.43 | 1.55 | 27.54 | 0.67 | 76.56 | 17 | |
| | | overall | 15.74 | 18.98 | 8.43 | 1.75 | 22.8 | 0.67 | 76.56 | 25 | |
| | 3rd | Control | 14.55 | 9.7 | 12.99 | 10.9 | 19.28 | 1.79 | 27.79 | 5 | 0.5663 |
| | | Patient | 26.63 | 30.57 | 21.02 | 2.68 | 25.51 | 0.84 | 109.31 | 13 | |
| | | overall | 23.28 | 26.7 | 17.24 | 4.74 | 25.32 | 0.84 | 109.31 | 18 | |
| CEFT | BL | Control | 58.62 | 37.01 | 53.87 | 40.23 | 68.26 | 13.34 | 134.64 | 8 | 0.0003 |
| Proliferation | | Patient | 13.91 | 14.55 | 8.55 | 1.57 | 24.59 | 1.13 | 45.2 | 17 | |
| | | overall | 28.21 | 31.52 | 21.69 | 2.61 | 45.2 | 1.13 | 134.64 | 25 | |
| | 2nd | Control | 44.37 | 23.29 | 45.27 | 23.57 | 59.98 | 14.02 | 77.59 | 8 | 0.0005 |
| | | Patient | 12.45 | 14.06 | 5.3 | 2 | 18.81 | 0.84 | 45.1 | 17 | |
| | | overall | 22.66 | 22.82 | 14.02 | 4.38 | 37.69 | 0.84 | 77.59 | 25 | |
| | 3rd | Control | 42.47 | 30.4 | 47.97 | 18.49 | 57.59 | 6.36 | 81.95 | 5 | 0.3359 |
| | | Patient | 26.98 | 21.9 | 17.3 | 10.99 | 35.4 | 2.4 | 66.92 | 13 | |
| | | overall | 31.28 | 24.64 | 18.41 | 11.57 | 53.43 | 2.4 | 81.95 | 18 | |
| PMA/iono | BL | Control | 239 | 104.88 | 263.19 | 183.08 | 309.21 | 85.41 | 374.98 | 8 | 0.0109 |
| Proliferation | | Patient | 131.44 | 125.23 | 72.24 | 64.81 | 153.72 | 40.28 | 532.1 | 17 | |
| | | overall | 165.86 | 127.62 | 94.69 | 67.64 | 255.1 | 40.28 | 532.1 | 25 | |
| | 2nd | Control | 202.67 | 145.51 | 152.03 | 86.43 | 359.89 | 51.72 | 392.65 | 8 | 0.4063 |
| | | Patient | 141.43 | 96.85 | 105.33 | 76.72 | 178.32 | 40.67 | 379.03 | 17 | |
| | | overall | 161.03 | 115.24 | 129.72 | 76.72 | 208.53 | 40.67 | 392.65 | 25 | |
| | 3rd | Control | 184.14 | 55.05 | 220.44 | 152.04 | 222 | 102.06 | 224.18 | 5 | 0.0194 |
| | | Patient | 113.74 | 147.45 | 60.92 | 43.21 | 113.89 | 21.89 | 586.07 | 13 | |
| | | overall | 133.3 | 130.81 | 105.47 | 48.56 | 150.11 | 21.89 | 586.07 | 18 | |
| IFNγ | BL | Control | 3.31 | 2.11 | 2.81 | 1.52 | 4.68 | 1.34 | 7.03 | 8 | 0.4063 |
| | | Patient | 32.97 | 60.05 | 5.36 | 1.25 | 44.35 | 0.44 | 233.91 | 17 | |
| | | overall | 23.48 | 51.04 | 4.65 | 1.39 | 8.47 | 0.44 | 233.91 | 25 | |
| | 2nd | Control | 15.96 | 20.83 | 8.79 | 4.66 | 16.14 | 1.91 | 65.06 | 8 | 0.1747 |

| | | | | | | | | | | |
|---|---|---|---|---|---|---|---|---|---|---|
| | | Patient | 45.79 | 96.92 | 2.27 | 1.5 | 5.95 | 0.45 | 359.61 | 17 | |
| | | overall | 36.24 | 81.18 | 5.2 | 1.91 | 14.71 | 0.45 | 359.61 | 25 | |
| | 3rd | Control | 26.58 | 24.42 | 19.89 | 10.49 | 38.58 | 1.33 | 62.63 | 5 | 0.6331 |
| | | Patient | 73.93 | 99.16 | 48.64 | 3 | 92.06 | 0.76 | 327.85 | 13 | |
| | | overall | 60.78 | 86.94 | 29.24 | 3.05 | 68.5 | 0.76 | 327.85 | 18 | |
| IL2 | BL | Control | 1.14 | 0.58 | 1 | 0.76 | 1.27 | 0.6 | 2.35 | 8 | 0.0266 |
| | | Patient | 16.6 | 35.76 | 4.91 | 1.11 | 10.77 | 0.61 | 146.61 | 17 | |
| | | overall | 11.65 | 30.11 | 1.19 | 0.94 | 6.36 | 0.6 | 146.61 | 25 | |
| | 2nd | Control | 3.08 | 2.55 | 2.47 | 1.33 | 3.37 | 0.97 | 8.71 | 8 | 0.2622 |
| | | Patient | 18.27 | 34.33 | 8.1 | 1.31 | 16.35 | 0.41 | 142.96 | 17 | |
| | | overall | 13.41 | 28.98 | 3.04 | 1.31 | 14.28 | 0.41 | 142.96 | 25 | |
| | 3rd | Control | 10.71 | 17.32 | 4.1 | 2.23 | 4.66 | 1 | 41.59 | 5 | 0.8490 |
| | | Patient | 14.63 | 24.83 | 5.51 | 1.96 | 16.88 | 0.68 | 93.37 | 13 | |
| | | overall | 13.54 | 22.56 | 4.38 | 2.02 | 16.38 | 0.68 | 93.37 | 18 | |
| IL10 | BL | Control | 1.21 | 0.23 | 1.22 | 1.18 | 1.24 | 0.8 | 1.63 | 8 | 0.2770 |
| | | Patient | 5.87 | 7.96 | 1.95 | 1 | 7.78 | 1 | 28.53 | 17 | |
| | | overall | 4.38 | 6.87 | 1.22 | 1 | 3.64 | 0.8 | 28.53 | 25 | |
| | 2nd | Control | 2.89 | 2.84 | 1.37 | 1.2 | 3.47 | 0.95 | 8.92 | 8 | 0.9302 |
| | | Patient | 6.65 | 14.47 | 1.74 | 1 | 4.34 | 0.26 | 61.22 | 17 | |
| | | overall | 5.44 | 12.05 | 1.51 | 1 | 4.34 | 0.26 | 61.22 | 25 | |
| | 3rd | Control | 2.29 | 0.9 | 2.06 | 1.74 | 3.11 | 1.22 | 3.31 | 5 | 0.7670 |
| | | Patient | 23.51 | 26.22 | 6.9 | 1 | 39.92 | 0.14 | 63.23 | 13 | |
| | | overall | 17.61 | 24.1 | 2.59 | 1.05 | 35.86 | 0.14 | 63.23 | 18 | |
| IL13 | BL | Control | 0.63 | 0.08 | 0.61 | 0.61 | 0.61 | 0.57 | 0.84 | 8 | 0.0001 |
| | | Patient | 16.04 | 28.93 | 1 | 1 | 20.62 | 0.96 | 98.93 | 17 | |
| | | overall | 11.11 | 24.73 | 1 | 0.61 | 2.87 | 0.57 | 98.93 | 25 | |
| | 2nd | Control | 2.7 | 2.05 | 2.07 | 1.08 | 4.02 | 0.6 | 5.89 | 8 | 0.2779 |
| | | Patient | 29.36 | 58.55 | 5.61 | 1 | 18.35 | 0.96 | 184.11 | 17 | |
| | | overall | 20.83 | 49.48 | 2.16 | 1 | 11.27 | 0.6 | 184.11 | 25 | |
| | 3rd | Control | 3.83 | 2.45 | 3.41 | 2.95 | 4.97 | 0.61 | 7.21 | 5 | 0.1390 |
| | | Patient | 44.51 | 51.03 | 32.26 | 1 | 66.93 | 0.53 | 153.83 | 13 | |
| | | overall | 33.21 | 46.81 | 8.44 | 1.49 | 40.64 | 0.53 | 153.83 | 18 | |
| TNFα | BL | Control | 0.87 | 0.54 | 0.71 | 0.56 | 1.18 | 0.28 | 1.93 | 8 | 0.0289 |
| | | Patient | 7.54 | 15.16 | 1.43 | 1 | 3.97 | 0.29 | 47.44 | 17 | |
| | | overall | 5.41 | 12.78 | 1.19 | 0.73 | 1.94 | 0.28 | 47.44 | 25 | |
| | 2nd | Control | 1.13 | 0.55 | 0.94 | 0.76 | 1.36 | 0.52 | 2.08 | 8 | 0.3665 |
| | | Patient | 5.79 | 8.43 | 1.48 | 0.73 | 3.54 | 0.2 | 23.19 | 17 | |
| | | overall | 4.3 | 7.24 | 1.1 | 0.76 | 2.64 | 0.2 | 23.19 | 25 | |
| | 3rd | Control | 1.2 | 0.38 | 1.32 | 1.28 | 1.35 | 0.55 | 1.51 | 5 | 0.1674 |
| | | Patient | 8.29 | 9.52 | 3.42 | 1.09 | 12.68 | 0.51 | 27.1 | 13 | |
| | | overall | 6.32 | 8.64 | 1.91 | 1.14 | 6.74 | 0.51 | 27.1 | 18 | |
| GM-CSF | BL | Control | 0.69 | 0.23 | 0.69 | 0.57 | 0.86 | 0.32 | 1 | 8 | 0.0109 |
| | | Patient | 13.11 | 26.82 | 1.33 | 0.94 | 13.77 | 0.33 | 111.87 | 17 | |
| | | overall | 9.13 | 22.68 | 1 | 0.69 | 8.63 | 0.32 | 111.87 | 25 | |
| | 2nd | Control | 3.06 | 2.74 | 2.17 | 1.4 | 3.48 | 0.65 | 8.83 | 8 | 0.5875 |
| | | Patient | 18.16 | 33.84 | 3.68 | 1 | 9.26 | 0.34 | 109.31 | 17 | |
| | | overall | 13.33 | 28.59 | 2.61 | 1 | 8.77 | 0.34 | 109.31 | 25 | |

| | | | | | | | | | | | |
|---|---|---|---|---|---|---|---|---|---|---|---|
| | 3rd | Control | 4.49 | 5.63 | 1.68 | 1.38 | 4.57 | 0.63 | 14.21 | 5 | 0.2460 |
| | | Patient | 22.33 | 33.7 | 10.33 | 1.03 | 28.94 | 0.63 | 126.68 | 13 | |
| | | overall | 17.38 | 29.61 | 6.82 | 1.11 | 24.68 | 0.63 | 126.68 | 18 | |

**Table S5.** The following table shows descriptive statistics for cellular data separately for humoral responders and non-responders (response was defined as an NT ≥ 10 and at least two-fold increase in titers after two vaccinations). The p-values give the result for the Wilcoxon-Test for the comparison between responders and non-responders.

| Variable | Time | Responder | Mean | SD | Median | Q1 | Q3 | Min | Max | N | p-value |
|---|---|---|---|---|---|---|---|---|---|---|---|
| Age | BL | No | 41.09 | 14.02 | 43 | 26.5 | 54 | 22 | 58 | 11 | 0.4493 |
| | | Yes | 32.33 | 14.33 | 27 | 26.25 | 30 | 22 | 61 | 6 | |
| | | overall | 38 | 14.34 | 31 | 26 | 53 | 22 | 61 | 17 | |
| BMI | BL | No | 28.55 | 5.86 | 27.8 | 24.1 | 30.7 | 22.7 | 43.1 | 11 | 0.2275 |
| | | Yes | 25.53 | 7.54 | 23.25 | 20.4 | 27.6 | 19.1 | 39.1 | 6 | |
| | | overall | 27.48 | 6.44 | 26.9 | 23.1 | 29.9 | 19.1 | 43.1 | 17 | |
| TBE | BL | No | 2.91 | 2.57 | 1.53 | 1.32 | 3.75 | 1.02 | 9.42 | 11 | 0.0002 |
| Proliferation | | Yes | 32.33 | 19.35 | 27.93 | 25.11 | 30.35 | 13.06 | 69.7 | 6 | |
| | | overall | 13.29 | 18.2 | 4.15 | 1.45 | 24.34 | 1.02 | 69.7 | 17 | |
| | 2nd | No | 6.87 | 10.02 | 1.72 | 1.3 | 6.79 | 0.67 | 31.55 | 11 | 0.0019 |
| | | Yes | 39.35 | 23.25 | 35.84 | 23.98 | 50.33 | 12.66 | 76.56 | 6 | |
| | | overall | 18.33 | 22.08 | 8.43 | 1.55 | 27.54 | 0.67 | 76.56 | 17 | |
| | 3rd | No | 22.45 | 39.41 | 2.68 | 1.8 | 20.36 | 0.84 | 109.31 | 7 | 0.1807 |
| | | Yes | 31.52 | 18.06 | 23.77 | 21.46 | 38.21 | 14.8 | 63.05 | 6 | |
| | | overall | 26.63 | 30.57 | 21.02 | 2.68 | 25.51 | 0.84 | 109.31 | 13 | |
| CEFT | BL | No | 6.77 | 10.59 | 2.21 | 1.46 | 6.01 | 1.13 | 36.13 | 11 | 0.0048 |
| Proliferation | | Yes | 27 | 11.65 | 24.62 | 22.41 | 32.16 | 11.21 | 45.2 | 6 | |
| | | overall | 13.91 | 14.55 | 8.55 | 1.57 | 24.59 | 1.13 | 45.2 | 17 | |
| | 2nd | No | 4.86 | 5.49 | 2.79 | 1.48 | 5.26 | 0.84 | 19.44 | 11 | 0.0011 |
| | | Yes | 26.38 | 14.6 | 26.61 | 13.09 | 36.87 | 11.08 | 45.1 | 6 | |
| | | overall | 12.45 | 14.06 | 5.3 | 2 | 18.81 | 0.84 | 45.1 | 17 | |
| | 3rd | No | 15.26 | 9.69 | 13.34 | 10.84 | 17.82 | 2.4 | 33.76 | 7 | 0.1014 |
| | | Yes | 40.64 | 24.95 | 45.32 | 20.39 | 60.87 | 8.17 | 66.92 | 6 | |
| | | overall | 26.98 | 21.9 | 17.3 | 10.99 | 35.4 | 2.4 | 66.92 | 13 | |
| PMA/iono | BL | No | 151.74 | 150.64 | 72.24 | 65.54 | 172.21 | 40.28 | 532.1 | 11 | 0.6605 |
| Proliferation | | Yes | 94.23 | 47.26 | 72.52 | 62.97 | 118.79 | 54.37 | 171.94 | 6 | |
| | | overall | 131.44 | 125.23 | 72.24 | 64.81 | 153.72 | 40.28 | 532.1 | 17 | |
| | 2nd | No | 142.39 | 96.34 | 105.33 | 77.88 | 172.33 | 50.56 | 379.03 | 11 | 0.7325 |
| | | Yes | 139.68 | 107 | 111.54 | 57.89 | 190.79 | 40.67 | 317.11 | 6 | |
| | | overall | 141.43 | 96.85 | 105.33 | 76.72 | 178.32 | 40.67 | 379.03 | 17 | |
| | 3rd | No | 151.92 | 196.19 | 60.92 | 50.17 | 139.29 | 37.55 | 586.07 | 7 | 0.3660 |
| | | Yes | 69.2 | 39.6 | 66.14 | 39.49 | 103.93 | 21.89 | 113.89 | 6 | |
| | | overall | 113.74 | 147.45 | 60.92 | 43.21 | 113.89 | 21.89 | 586.07 | 13 | |
| IFNγ | BL | No | 8.3 | 17.96 | 1.39 | 0.92 | 6.65 | 0.44 | 61.7 | 11 | 0.0031 |
| | | Yes | 78.19 | 84.27 | 58.38 | 20.65 | 93.38 | 5.36 | 233.91 | 6 | |
| | | overall | 32.97 | 60.05 | 5.36 | 1.25 | 44.35 | 0.44 | 233.91 | 17 | |
| | 2nd | No | 16.45 | 48.65 | 1.57 | 1.33 | 2.25 | 0.45 | 163.11 | 11 | 0.0031 |
| | | Yes | 99.58 | 141.28 | 32.01 | 5.84 | 136.65 | 5.2 | 359.61 | 6 | |
| | | overall | 45.79 | 96.92 | 2.27 | 1.5 | 5.95 | 0.45 | 359.61 | 17 | |
| | 3rd | No | 44.31 | 78.82 | 3 | 2.47 | 47.63 | 0.76 | 206.18 | 7 | 0.1014 |
| | | Yes | 108.49 | 116.12 | 64.04 | 50.89 | 123.34 | 5.42 | 327.85 | 6 | |
| | | overall | 73.93 | 99.16 | 48.64 | 3 | 92.06 | 0.76 | 327.85 | 13 | |

| | | | | | | | | | | |
|---|---|---|---|---|---|---|---|---|---|---|
| IL2 | BL | No | 2.55 | 2.52 | 1.19 | 0.93 | 3.75 | 0.61 | 7.55 | 11 | 0.0006 |
| | | Yes | 42.35 | 53.37 | 20.07 | 12.71 | 43.25 | 6.12 | 146.61 | 6 | |
| | | overall | 16.6 | 35.76 | 4.91 | 1.11 | 10.77 | 0.61 | 146.61 | 17 | |
| | 2nd | No | 5.63 | 7.66 | 1.41 | 1.11 | 7.01 | 0.41 | 24.02 | 11 | 0.0071 |
| | | Yes | 41.45 | 51.56 | 18.04 | 14.8 | 40.38 | 8.1 | 142.96 | 6 | |
| | | overall | 18.27 | 34.33 | 8.1 | 1.31 | 16.35 | 0.41 | 142.96 | 17 | |
| | 3rd | No | 15.28 | 34.48 | 1.96 | 0.9 | 4.57 | 0.68 | 93.37 | 7 | 0.0734 |
| | | Yes | 13.87 | 7.21 | 15.87 | 8.95 | 18.98 | 3.34 | 21.46 | 6 | |
| | | overall | 14.63 | 24.83 | 5.51 | 1.96 | 16.88 | 0.68 | 93.37 | 13 | |
| IL10 | BL | No | 3.48 | 5.82 | 1 | 1 | 3.01 | 1 | 20.68 | 11 | 0.0352 |
| | | Yes | 10.27 | 9.97 | 8.78 | 3.4 | 11.78 | 1.14 | 28.53 | 6 | |
| | | overall | 5.87 | 7.96 | 1.95 | 1 | 7.78 | 1 | 28.53 | 17 | |
| | 2nd | No | 1.64 | 1.3 | 1 | 1 | 1.98 | 0.26 | 4.09 | 11 | 0.0029 |
| | | Yes | 15.83 | 22.6 | 7.13 | 4.61 | 12.25 | 1.74 | 61.22 | 6 | |
| | | overall | 6.65 | 14.47 | 1.74 | 1 | 4.34 | 0.26 | 61.22 | 17 | |
| | 3rd | No | 14.25 | 22.71 | 1 | 0.91 | 18.6 | 0.14 | 59.56 | 7 | 0.0985 |
| | | Yes | 34.31 | 27.76 | 38.82 | 10.6 | 56.81 | 1 | 63.23 | 6 | |
| | | overall | 23.51 | 26.22 | 6.9 | 1 | 39.92 | 0.14 | 63.23 | 13 | |
| IL13 | BL | No | 1.21 | 0.57 | 1 | 1 | 1 | 0.96 | 2.87 | 11 | 0.0006 |
| | | Yes | 43.21 | 36.16 | 23.64 | 20.75 | 65.66 | 13.63 | 98.93 | 6 | |
| | | overall | 16.04 | 28.93 | 1 | 1 | 20.62 | 0.96 | 98.93 | 17 | |
| | 2nd | No | 3.87 | 5.69 | 1 | 1 | 3.33 | 0.96 | 18.35 | 11 | 0.0017 |
| | | Yes | 76.09 | 82.8 | 33.08 | 18.4 | 144.39 | 11.27 | 184.11 | 6 | |
| | | overall | 29.36 | 58.55 | 5.61 | 1 | 18.35 | 0.96 | 184.11 | 17 | |
| | 3rd | No | 17.62 | 26.71 | 1 | 0.94 | 26.51 | 0.53 | 66.93 | 7 | 0.0381 |
| | | Yes | 75.89 | 56.58 | 69.99 | 32.32 | 112.44 | 15.16 | 153.83 | 6 | |
| | | overall | 44.51 | 51.03 | 32.26 | 1 | 66.93 | 0.53 | 153.83 | 13 | |
| TNFα | BL | No | 1.27 | 0.97 | 1.11 | 0.89 | 1.37 | 0.29 | 3.97 | 11 | 0.0022 |
| | | Yes | 19.04 | 22.1 | 7.16 | 4.09 | 37.39 | 1.94 | 47.44 | 6 | |
| | | overall | 7.54 | 15.16 | 1.43 | 1 | 3.97 | 0.29 | 47.44 | 17 | |
| | 2nd | No | 2.61 | 5.72 | 0.79 | 0.65 | 1.28 | 0.2 | 19.82 | 11 | 0.0042 |
| | | Yes | 11.63 | 9.93 | 8.84 | 3.21 | 20.93 | 2.64 | 23.19 | 6 | |
| | | overall | 5.79 | 8.43 | 1.48 | 0.73 | 3.54 | 0.2 | 23.19 | 17 | |
| | 3rd | No | 5.09 | 9.76 | 1.09 | 0.62 | 2.86 | 0.51 | 27.1 | 7 | 0.0535 |
| | | Yes | 12.02 | 8.49 | 9.95 | 5.76 | 19.77 | 2.69 | 22.13 | 6 | |
| | | overall | 8.29 | 9.52 | 3.42 | 1.09 | 12.68 | 0.51 | 27.1 | 13 | |
| GM-CSF | BL | No | 2.61 | 5.29 | 1 | 0.63 | 1.21 | 0.33 | 18.43 | 11 | 0.0011 |
| | | Yes | 32.35 | 39.49 | 18.79 | 12.18 | 24.22 | 8.63 | 111.87 | 6 | |
| | | overall | 13.11 | 26.82 | 1.33 | 0.94 | 13.77 | 0.33 | 111.87 | 17 | |
| | 2nd | No | 11.43 | 32.49 | 1.07 | 0.56 | 3.14 | 0.34 | 109.31 | 11 | 0.0048 |
| | | Yes | 30.5 | 35.66 | 16.57 | 8.89 | 33.01 | 5.68 | 99.37 | 6 | |
| | | overall | 18.16 | 33.84 | 3.68 | 1 | 9.26 | 0.34 | 109.31 | 17 | |
| | 3rd | No | 24.65 | 46.43 | 1.03 | 0.91 | 21.18 | 0.63 | 126.68 | 7 | 0.3660 |
| | | Yes | 19.64 | 11.07 | 22.08 | 9.65 | 28.52 | 6.39 | 31.08 | 6 | |
| | | overall | 22.33 | 33.7 | 10.33 | 1.03 | 28.94 | 0.63 | 126.68 | 13 | |

**Table S6.** The following table shows descriptive statistics for cellular data separately for patients with related and unrelated donor. The p-values give the result for the Wilcoxon-Test for the comparison between groups.

| Variable | Time | Donor | Mean | SD | Median | Q1 | Q3 | Min | Max | N | p.value |
|---|---|---|---|---|---|---|---|---|---|---|---|
| Age | BL | Non-related | 45.12 | 13.71 | 50 | 39 | 55.25 | 22 | 58 | 8 | 0.101 |
| | | related | 31.67 | 12.29 | 27 | 26 | 31 | 22 | 61 | 9 | |
| | | overall | 38 | 14.34 | 31 | 26 | 53 | 22 | 61 | 17 | |
| BMI | BL | Non-related | 29.36 | 5.29 | 29.15 | 26.62 | 31.73 | 22.7 | 39.1 | 8 | 0.178 |
| | | related | 25.81 | 7.2 | 24 | 23.1 | 26.9 | 19.1 | 43.1 | 9 | |
| | | overall | 27.48 | 6.44 | 26.9 | 23.1 | 29.9 | 19.1 | 43.1 | 17 | |
| TBE | BL | Non-related | 4.8 | 9.57 | 1.45 | 1.17 | 1.7 | 1.02 | 28.45 | 8 | 0.004 |
| Proliferation | | related | 20.84 | 21.12 | 13.06 | 5.15 | 27.41 | 3.35 | 69.7 | 9 | |
| | | overall | 13.29 | 18.2 | 4.15 | 1.45 | 24.34 | 1.02 | 69.7 | 17 | |
| | 2nd | Non-related | 8.27 | 17.89 | 1.6 | 1 | 3.48 | 0.67 | 52.4 | 8 | 0.015 |
| | | related | 27.28 | 22.45 | 22.8 | 12.66 | 31.55 | 1.55 | 76.56 | 9 | |
| | | overall | 18.33 | 22.08 | 8.43 | 1.55 | 27.54 | 0.67 | 76.56 | 17 | |
| | 3rd | Non-related | 4.38 | 5.86 | 2.07 | 1.52 | 2.68 | 0.84 | 14.8 | 5 | 0.002 |
| | | related | 40.54 | 31.74 | 25.13 | 22.35 | 47.78 | 15.21 | 109.31 | 8 | |
| | | overall | 26.63 | 30.57 | 21.02 | 2.68 | 25.51 | 0.84 | 109.31 | 13 | |
| CEFT | BL | Non-related | 9.48 | 12.83 | 2.84 | 1.35 | 11.83 | 1.13 | 36.13 | 8 | 0.167 |
| Proliferation | | related | 17.85 | 15.57 | 14.59 | 2.61 | 24.65 | 1.53 | 45.2 | 9 | |
| | | overall | 13.91 | 14.55 | 8.55 | 1.57 | 24.59 | 1.13 | 45.2 | 17 | |
| | 2nd | Non-related | 3.86 | 3.48 | 3.19 | 1.08 | 5.23 | 0.84 | 11.08 | 8 | 0.011 |
| | | related | 20.09 | 15.68 | 18.81 | 9.51 | 34.41 | 1.84 | 45.1 | 9 | |
| | | overall | 12.45 | 14.06 | 5.3 | 2 | 18.81 | 0.84 | 45.1 | 17 | |
| | 3rd | Non-related | 16.44 | 11.27 | 15.38 | 13.34 | 17.3 | 2.4 | 33.76 | 5 | 0.354 |
| | | related | 33.56 | 24.92 | 26.87 | 10.91 | 57.12 | 8.17 | 66.92 | 8 | |
| | | overall | 26.98 | 21.9 | 17.3 | 10.99 | 35.4 | 2.4 | 66.92 | 13 | |
| PMA/iono | BL | Non-related | 117.12 | 93.64 | 71.89 | 65.91 | 118.69 | 54.37 | 322.35 | 8 | 1.000 |
| Proliferation | | related | 144.17 | 152.67 | 77.4 | 61.41 | 153.72 | 40.28 | 532.1 | 9 | |
| | | overall | 131.44 | 125.23 | 72.24 | 64.81 | 153.72 | 40.28 | 532.1 | 17 | |
| | 2nd | Non-related | 107.21 | 57.05 | 90.51 | 71.84 | 123.58 | 50.56 | 208.53 | 8 | 0.370 |
| | | related | 171.86 | 117.05 | 140.33 | 85.5 | 231.46 | 40.67 | 379.03 | 9 | |
| | | overall | 141.43 | 96.85 | 105.33 | 76.72 | 178.32 | 40.67 | 379.03 | 17 | |
| | 3rd | Non-related | 79.79 | 56.18 | 60.92 | 37.55 | 134.27 | 21.89 | 144.31 | 5 | 0.833 |
| | | related | 134.96 | 184.74 | 71.23 | 46.01 | 110.14 | 38.25 | 586.07 | 8 | |
| | | overall | 113.74 | 147.45 | 60.92 | 43.21 | 113.89 | 21.89 | 586.07 | 13 | |
| IFNγ | BL | Non-related | 11.52 | 24.75 | 1.89 | 1.14 | 5.86 | 0.44 | 72.41 | 8 | 0.139 |
| | | related | 52.04 | 76.2 | 12.75 | 5.36 | 61.7 | 0.54 | 233.91 | 9 | |
| | | overall | 32.97 | 60.05 | 5.36 | 1.25 | 44.35 | 0.44 | 233.91 | 17 | |
| | 2nd | Non-related | 21.66 | 57.05 | 1.47 | 1.29 | 2.24 | 0.45 | 162.84 | 8 | 0.021 |
| | | related | 67.24 | 121.82 | 5.81 | 3.68 | 58.07 | 1.5 | 359.61 | 9 | |
| | | overall | 45.79 | 96.92 | 2.27 | 1.5 | 5.95 | 0.45 | 359.61 | 17 | |
| | 3rd | Non-related | 67.35 | 145.63 | 2.79 | 2.16 | 3.19 | 0.76 | 327.85 | 5 | 0.127 |
| | | related | 78.04 | 68.47 | 64.04 | 37.84 | 104.29 | 3 | 206.18 | 8 | |
| | | overall | 73.93 | 99.16 | 48.64 | 3 | 92.06 | 0.76 | 327.85 | 13 | |
| IL2 | BL | Non-related | 2.9 | 3.48 | 1.19 | 1.05 | 3.16 | 0.61 | 10.77 | 8 | 0.059 |

| | | | | | | | | | | | |
|---|---|---|---|---|---|---|---|---|---|---|---|
| | | related | 28.77 | 46.83 | 7.55 | 6.12 | 21.6 | 0.66 | 146.61 | 9 | |
| | | overall | 16.6 | 35.76 | 4.91 | 1.11 | 10.77 | 0.61 | 146.61 | 17 | |
| | 2nd | Non-related | 3.65 | 6.55 | 1.24 | 1.01 | 1.88 | 0.41 | 19.73 | 8 | 0.004 |
| | | related | 31.26 | 43.78 | 14.72 | 10.75 | 24.02 | 2.92 | 142.96 | 9 | |
| | | overall | 18.27 | 34.33 | 8.1 | 1.31 | 16.35 | 0.41 | 142.96 | 17 | |
| | 3rd | Non-related | 4.82 | 8.32 | 1.05 | 0.75 | 1.96 | 0.68 | 19.68 | 5 | 0.045 |
| | | related | 20.75 | 30.1 | 10.92 | 5.04 | 18.03 | 3.34 | 93.37 | 8 | |
| | | overall | 14.63 | 24.83 | 5.51 | 1.96 | 16.88 | 0.68 | 93.37 | 13 | |
| IL10 | BL | Non-related | 2.99 | 3.94 | 1.22 | 1 | 2.7 | 1 | 12.45 | 8 | 0.184 |
| | | related | 8.44 | 9.88 | 4.1 | 1.14 | 9.79 | 1 | 28.53 | 9 | |
| | | overall | 5.87 | 7.96 | 1.95 | 1 | 7.78 | 1 | 28.53 | 17 | |
| | 2nd | Non-related | 2 | 1.82 | 1 | 0.93 | 3.04 | 0.26 | 5.41 | 8 | 0.053 |
| | | related | 10.78 | 19.37 | 4.09 | 1.19 | 8.85 | 1 | 61.22 | 9 | |
| | | overall | 6.65 | 14.47 | 1.74 | 1 | 4.34 | 0.26 | 61.22 | 17 | |
| | 3rd | Non-related | 9.32 | 16.11 | 1 | 0.83 | 6.9 | 0.14 | 37.72 | 5 | 0.091 |
| | | related | 32.38 | 28.22 | 35.11 | 1.42 | 60.28 | 1 | 63.23 | 8 | |
| | | overall | 23.51 | 26.22 | 6.9 | 1 | 39.92 | 0.14 | 63.23 | 13 | |
| IL13 | BL | Non-related | 3.51 | 6.92 | 1 | 1 | 1.12 | 0.96 | 20.62 | 8 | 0.042 |
| | | related | 27.17 | 36.55 | 13.63 | 1 | 26.13 | 1 | 98.93 | 9 | |
| | | overall | 16.04 | 28.93 | 1 | 1 | 20.62 | 0.96 | 98.93 | 17 | |
| | 2nd | Non-related | 4.64 | 10.28 | 1 | 1 | 1.01 | 0.96 | 30.08 | 8 | 0.011 |
| | | related | 51.33 | 74.9 | 14.51 | 10.59 | 36.09 | 1 | 184.11 | 9 | |
| | | overall | 29.36 | 58.55 | 5.61 | 1 | 18.35 | 0.96 | 184.11 | 17 | |
| | 3rd | Non-related | 7.13 | 14.05 | 1 | 0.88 | 1 | 0.53 | 32.26 | 5 | 0.010 |
| | | related | 67.88 | 52.25 | 55.14 | 28.18 | 109.12 | 9.68 | 153.83 | 8 | |
| | | overall | 44.51 | 51.03 | 32.26 | 1 | 66.93 | 0.53 | 153.83 | 13 | |
| TNFα | BL | Non-related | 1.83 | 2.16 | 1.25 | 0.97 | 1.43 | 0.29 | 7.08 | 8 | 0.194 |
| | | related | 12.62 | 19.85 | 3.1 | 1.11 | 7.23 | 0.46 | 47.44 | 9 | |
| | | overall | 7.54 | 15.16 | 1.43 | 1 | 3.97 | 0.29 | 47.44 | 17 | |
| | 2nd | Non-related | 2.39 | 4.75 | 0.76 | 0.59 | 1 | 0.2 | 14.13 | 8 | 0.014 |
| | | related | 8.82 | 10.02 | 3.1 | 1.76 | 19.82 | 0.7 | 23.19 | 9 | |
| | | overall | 5.79 | 8.43 | 1.48 | 0.73 | 3.54 | 0.2 | 23.19 | 17 | |
| | 3rd | Non-related | 3.1 | 5.36 | 0.72 | 0.52 | 1.09 | 0.51 | 12.68 | 5 | 0.034 |
| | | related | 11.53 | 10.38 | 6.25 | 3.24 | 22.13 | 2.3 | 27.1 | 8 | |
| | | overall | 8.29 | 9.52 | 3.42 | 1.09 | 12.68 | 0.51 | 27.1 | 13 | |
| GM-CSF | BL | Non-related | 3.84 | 8.3 | 1.05 | 0.84 | 1.15 | 0.33 | 24.36 | 8 | 0.114 |
| | | related | 21.35 | 34.87 | 11.65 | 2.82 | 18.43 | 0.44 | 111.87 | 9 | |
| | | overall | 13.11 | 26.82 | 1.33 | 0.94 | 13.77 | 0.33 | 111.87 | 17 | |
| | 2nd | Non-related | 5.22 | 12.47 | 0.78 | 0.52 | 1.25 | 0.34 | 36.05 | 8 | 0.004 |
| | | related | 29.67 | 42.88 | 8.77 | 4.44 | 23.88 | 2.61 | 109.31 | 9 | |
| | | overall | 18.16 | 33.84 | 3.68 | 1 | 9.26 | 0.34 | 109.31 | 17 | |
| | 3rd | Non-related | 4.07 | 7.16 | 1.01 | 0.81 | 1.03 | 0.63 | 16.88 | 5 | 0.011 |
| | | related | 33.75 | 39.12 | 28.11 | 9.56 | 31.32 | 6.39 | 126.68 | 8 | |
| | | overall | 22.33 | 33.7 | 10.33 | 1.03 | 28.94 | 0.63 | 126.68 | 13 | |

**Figure S1:** Boxplots showing the median and distribution of lymphocyte proliferation as detected by thymidine incorporation assay (the stimulation indices are given) at three different time points for healthy controls (orange) and patients (green) after stimulation with CEFT antigen (CMV, EBV, influenza virus and Clostridium tetani antigen mix). Asterisk marks significant p-value (Baseline: $p < 0.001$; 2nd vaccination: $p < 0.001$).

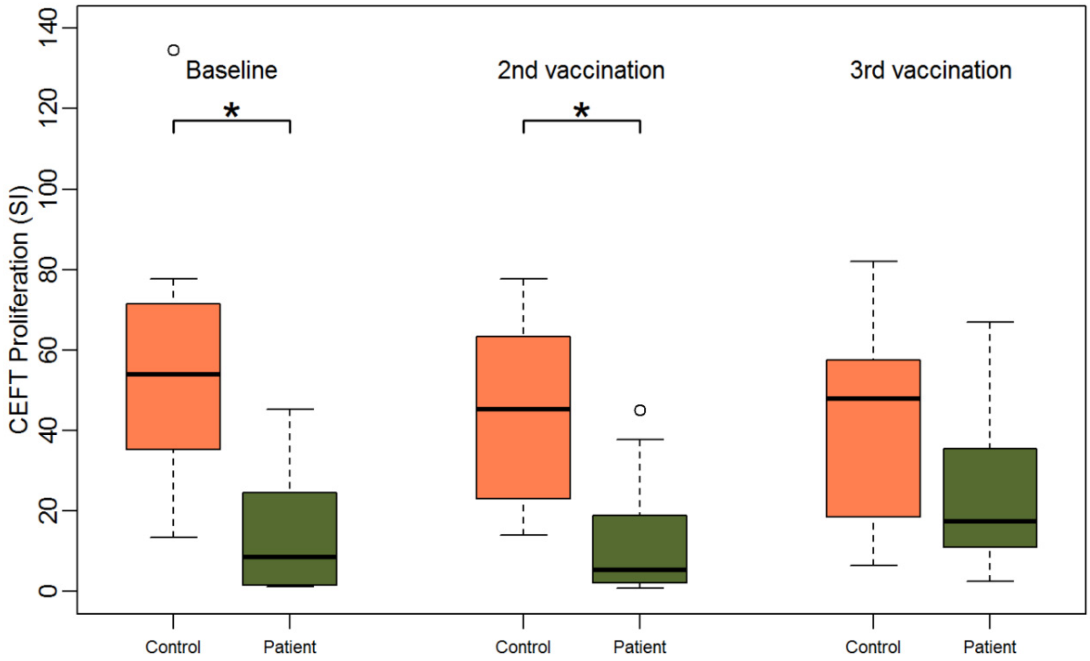

**Figure S2:** Boxplots showing the median and distribution of lymphocyte proliferation as detected by thymidine incorporation assay (the stimulation indices are given) at three different time points for healthy controls (orange) and patients (green) after stimulation with PMA (phorphole myristate acetate plus ionomycin). Asterisk marks significant p-value (Baseline: $p=0.01$; 3rd vaccination: $p=0.02$).

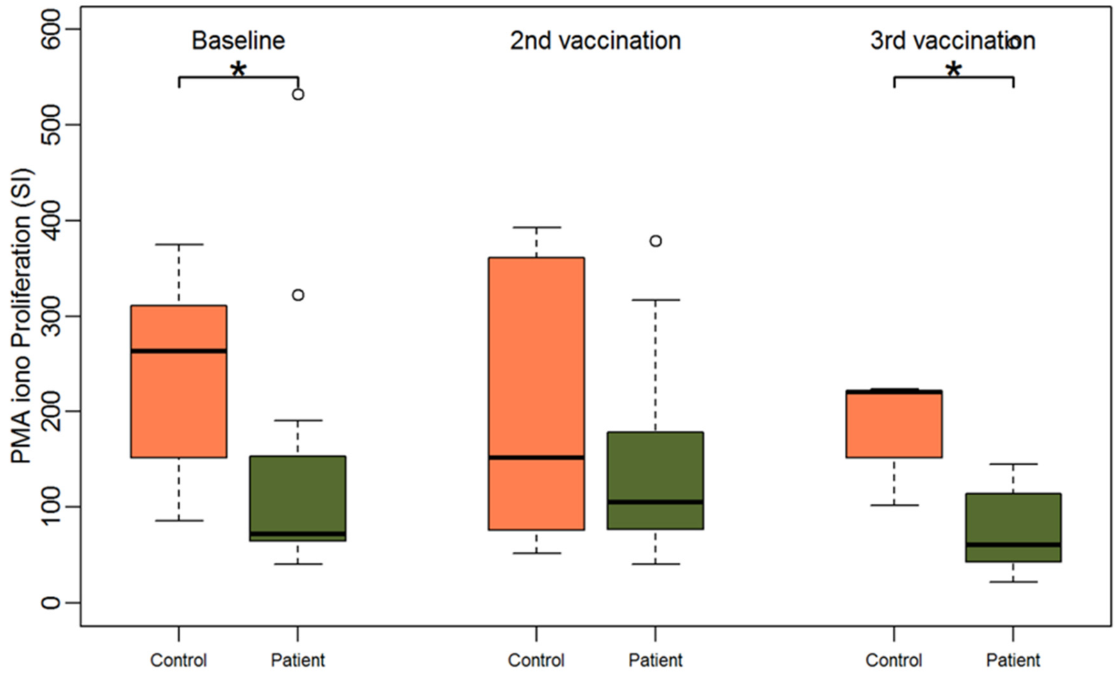

**Figure S3:** Boxplots showing the median and distribution of lymphocyte proliferation as detected by thymidine incorporation assay (the stimulation indices are given) at three different time points for humoral non-responders (orange) and responders (green) after stimulation with CEFT antigen (CMV, EBV, influenza virus and Clostridium tetani antigen mix). Asterisk marks significant p-value (Baseline: p=0.005; 2nd vaccination: p= 0.001).

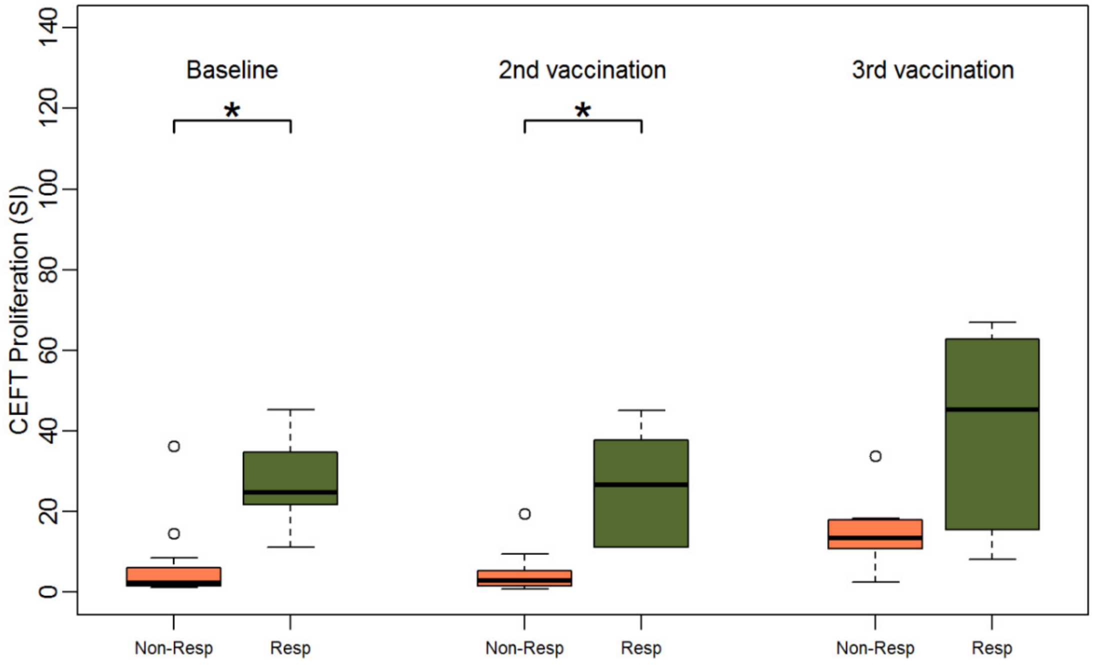

**Figure S4:** Boxplots showing the median and distribution of lymphocyte proliferation as detected by thymidine incorporation assay (the stimulation indices are given) at three different time points for humoral non-responders (orange) and responders (green) after stimulation with PMA (phorphole myristate acetate plus ionomycin).

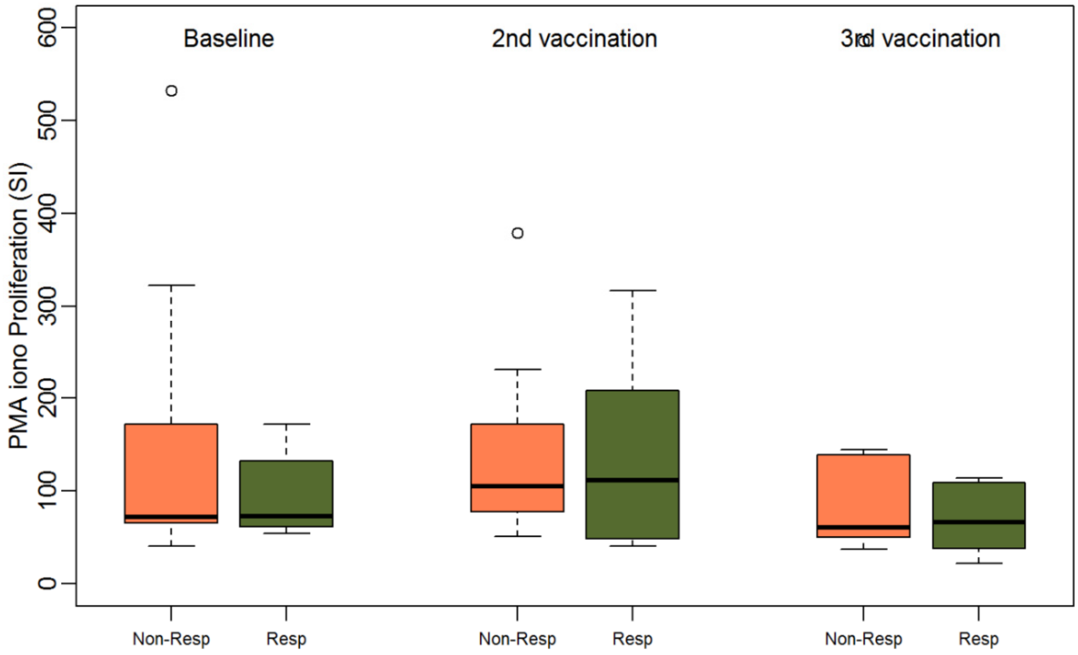

**Figure S5:** Correlation plot showing a significant correlation between TBE (tick-borne encephalitis antigen) lymphocyte proliferation and CEFT (CMV, EBV, influenza virus and Clostridium tetani antigen mix) lymphocyte proliferation values at baseline for patients (p=0.001, correlation coefficient: 0.71).

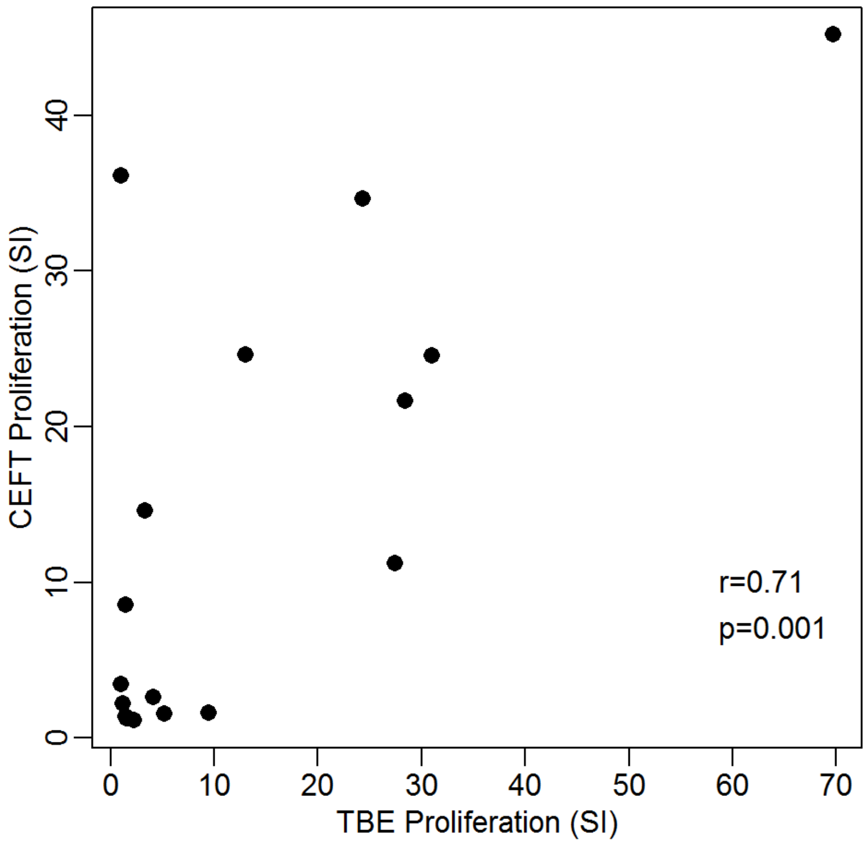

Supplement: Supplementary file 1 [file vaccines-09-00908-s001.zip › vaccines-1312457-supplementary.pdf]
